# Meeting of Societies

**Published:** 1937

**Authors:** 


### Meetings of Societies

THE Annual Meeting of the Society was held in the Physiological Lecture Theatre of the University on Wednesday, 13th October, at 8.30 p.m.

Dr. Richard Clarke was in the Chair, and eighty-five members were present.

The minutes of the previous meeting were read and confirmed.

The President then resigned his Chair to his successor, Mr. C. A. Moore. A vote of thanks to the retiring President was proposed by Dr. Symes, and seconded by Mr. Chitty.

The President gave his inaugural address on "**Medical Defence.**" A vote of thanks to the President for his address was proposed by Mr. Walters, and seconded by Dr. Orr-Ewing.

The annual reports of the Hon. Secretary, the Hon. Treasurer, and the Hon. Editor of the *Journal* were read and adopted by the Society, and Mr. R. V. Cooke and Mr. A. E. Iles were re-elected as Hon. Secretary and Hon. Treasurer respectively.

Dr. Nixon proposed and Dr. Richard Clarke seconded that the election of Hon. Medical Librarian should be postponed, and that the Society should elect the Professors of Medicine, Surgery and Gynæcology to act in the capacity of Librarian for the time being. This was adopted.

Dr. Elwin Harris proposed and Dr. Cookson seconded that the following nine members should serve on the Committee: Dr. Carleton, Dr. Corfield, Dr. Richard Clarke, Dr. Taylor, Dr. Martin, Dr. Milling, Mr. Wood, Mr. Shepherd and Mr. Jackman, and the following three members were asked to serve on the University Library Sub-Committee: Professor Perry, Professor Rendle Short, and Professor Drew Smythe.

---

The Second Meeting of the Session was held on Wednesday, 10th November, at 8.30 p.m. in the Physiological Lecture Theatre of the University.

### BRISTOL MEDICO-CHIRURGICAL SOCIETY.

### INCOME AND EXPENDITURE ACCOUNT for the year ended 30th September, 1937.

**INCOME.**

| | £ | s. | d. | £ | s. | d. |
|---|---|---|---|---|---|---|
| Members' Subscriptions .. | 227 | 6 | 6 | | | |
| Do., outstanding for 1936–1937 .. .. .. .. .. | 29 | 8 | 0 | | | |
| | | | | 256 | 14 | 6 |
| Interest on Bank Deposits .. .. | | | | 2 | 11 | 0 |
| Interest on £500 4 per cent. Consolidated Stock, less tax .. .. | | | | 15 | 2 | 6 |
| | | | | 274 | 8 | 0 |
| Deficit for the year .. .. .. .. | | | | 65 | 3 | 5 |
| | | | | £339 | 11 | 5 |

**EXPENDITURE.**

| | £ | s. | d. | £ | s. | d. | £ | s. | d. |
|---|---|---|---|---|---|---|---|---|---|
| Grant to *The Bristol Medico-Chirurgical Journal* .. .. .. .. | | | | | | | 261 | 0 | 0 |
| Librarian—Honorarium .. .. .. .. | | | | | | | 15 | 0 | 0 |
| Miscellaneous Expenses— | | | | | | | | | |
| Goodway–Refreshments | | | | 16 | 16 | 0 | | | |
| Postages and Sundries | | | | 13 | 2 | 2 | | | |
| Printing and Stationery | | | | 10 | 7 | 6 | | | |
| Audit Fee .. .. .. .. | | | | 2 | 2 | 0 | | | |
| Lantern and Cinematograph at Meetings .. | | | | 4 | 4 | 0 | | | |
| Electric Pointer .. .. | | | | 9 | 10 | 4 | | | |
| University Marshal .. | | | | 1 | 0 | 0 | | | |
| Lewis's Medical and Scientific Library— | | | | | | | | | |
| Subscription | 5 | 10 | 0 | | | | | | |
| Postages .. | 0 | 19 | 5 | | | | | | |
| | | | | 6 | 9 | 5 | | | |
| | | | | | | | 63 | 11 | 5 |
| | | | | | | | £339 | 11 | 5 |

### BALANCE SHEET as at 30th September, 1937.

**LIABILITIES AND CAPITAL.**

| | £ | s. | d. | £ | s. | d. |
|---|---|---|---|---|---|---|
| Sundry Creditors .. .. | | | | 7 | 2 | 3 |
| Capital as at 30th Sept., 1936 .. .. .. .. | 1,132 | 17 | 3 | | | |
| *Less* Deficit for the year | 65 | 3 | 5 | | | |
| | | | | 1,067 | 13 | 10 |
| | | | | £1,074 | 16 | 1 |

**ASSETS.**

| | £ | s. | d. | £ | s. | d. |
|---|---|---|---|---|---|---|
| Cash at Bank— | | | | | | |
| Current Account .. .. | 434 | 5 | 9 | | | |
| Deposit Account .. .. | 173 | 13 | 4 | | | |
| | | | | 607 | 19 | 1 |
| £500 4 per cent. Consolidated Stock at cost .. .. .. .. .. .. | | | | 437 | 9 | 0 |
| Sundry Debtors— Members' Subscriptions for 1936-1937 outstanding .. | | | | 29 | 8 | 0 |
| | | | | £1,074 | 16 | 1 |

Audited and found correct,

H. E. KEELER,

*Chartered Accountant,*

*Auditor.*

16–18 Clare Street, Bristol 1.

11*th October*, 1937.

Mr. C. A. Moore was in the Chair, and sixty-seven members present.

The minutes of the previous meeting were read and confirmed.

Dr. Barbour gave an address on "**Child Guidance.**"

---

The third meeting of the Session was held at 8.30 p.m. on Wednesday, 8th December, in the Physiology Lecture Theatre of the University.

Mr. C. A. Moore was in the Chair, and sixty-one members were present.

The minutes of the previous meeting were read and confirmed.

Mr. John Wright opened a discussion on "**The Nature and Treatment of Giddiness.**" He was followed by Dr. Carleton, Mr. E. Watson-Williams, Mr. Scarff and Dr. P. Watson-Williams.

---

### ℞ Galenicals ℞

*Hon. President:* Professor T. B. Davie, M.R.C.P.
*Hon. Secretary:* D. C. Bodenham,
Greystoke, South Dene, Coombe Lane, Bristol.

DURING 1937 the Society was honoured by the visits of many notable speakers, including Sir Humphry Rolleston, G.C.V.O., K.C.B., who gave a lecture entitled "**Medical Eponym**"; J. R. Rees, M.D., a lecture on "**Psychology**"; Sir Henry Dale, C.B.E., M.D., F.R.C.P., a lecture on "**The History of Knowledge concerning Ergot and its action.**" Professor Davie, M.R.C.P., exhibited a film by the late R. G. Canti, "**The Culture of Living Tissue.**"

A most successful trip to Germany was organized during the summer, a full account of which appeared in *The Black Bag*.

The Society has now over two hundred members, and the question of affiliation to the Bristol University Union is in hand.

